# Modulation of immune responses using adjuvants to facilitate therapeutic vaccination

**DOI:** 10.1111/imr.12889

**Published:** 2020-06-28

**Authors:** Virgil Schijns, Alberto Fernández‐Tejada, Žarko Barjaktarović, Ilias Bouzalas, Jens Brimnes, Sergey Chernysh, Sveinbjorn Gizurarson, Ihsan Gursel, Žiga Jakopin, Maria Lawrenz, Cristina Nativi, Stephane Paul, Gabriel Kristian Pedersen, Camillo Rosano, Ane Ruiz‐de‐Angulo, Bram Slütter, Aneesh Thakur, Dennis Christensen, Ed C. Lavelle

**Affiliations:** ^1^ Wageningen University, Cell Biology & Immunology and ERC‐The Netherlands Schaijk Landerd campus The Netherlands; ^2^ Chemical Immunology Lab Center for Cooperative Research in Biosciences CIC bioGUNE Biscay Spain; ^3^ Ikerbasque Basque Foundation for Science Bilbao Spain; ^4^ Agency for Medicines and Medical Devices of Montenegro Podgorica Montenegro; ^5^ Hellenic Agricultural Organization‐DEMETER, Veterinary Research Institute Thessaloniki Greece; ^6^ Alk Abello Copenhagen Denmark; ^7^ Laboratory of Insect Biopharmacology and Immunology Department of Entomology Saint‐Petersburg State University Saint‐Petersburg Russia; ^8^ Faculty of Pharmaceutical Sciences University of Iceland Reykjavik Iceland; ^9^ Bilkent University Ankara Turkey; ^10^ Faculty of Pharmacy University of Ljubljana Ljubljana Slovenia; ^11^ Vaccine Formulation Institute (CH) Geneva Switzerland; ^12^ Department of Chemistry University of Florence Florence Italy; ^13^ St Etienne University St Etienne France; ^14^ Statens Serum Institut Copenhagen Denmark; ^15^ IRCCS Policlinico San Martino Genova Italy; ^16^ Div. BioTherapeutics Leiden Academic Centre for Drug Research Leiden University Leiden The Netherlands; ^17^ University of Copenhagen Copenhagen Denmark; ^18^ Adjuvant Research Group School of Biochemistry and Immunology Trinity College Dublin Dublin Ireland

**Keywords:** adjuvant, autoimmunity, cancer, cellular immunity, therapeutic, vaccine

## Abstract

Therapeutic vaccination offers great promise as an intervention for a diversity of infectious and non‐infectious conditions. Given that most chronic health conditions are thought to have an immune component, vaccination can at least in principle be proposed as a therapeutic strategy. Understanding the nature of protective immunity is of vital importance, and the progress made in recent years in defining the nature of pathological and protective immunity for a range of diseases has provided an impetus to devise strategies to promote such responses in a targeted manner. However, in many cases, limited progress has been made in clinical adoption of such approaches. This in part results from a lack of safe and effective vaccine adjuvants that can be used to promote protective immunity and/or reduce deleterious immune responses. Although somewhat simplistic, it is possible to divide therapeutic vaccine approaches into those targeting conditions where antibody responses can mediate protection and those where the principal focus is the promotion of effector and memory cellular immunity or the reduction of damaging cellular immune responses as in the case of autoimmune diseases. Clearly, in all cases of antigen‐specific immunotherapy, the identification of protective antigens is a vital first step. There are many challenges to developing therapeutic vaccines beyond those associated with prophylactic diseases including the ongoing immune responses in patients, patient heterogeneity, and diversity in the type and stage of disease. If reproducible biomarkers can be defined, these could allow earlier diagnosis and intervention and likely increase therapeutic vaccine efficacy. Current immunomodulatory approaches related to adoptive cell transfers or passive antibody therapy are showing great promise, but these are outside the scope of this review which will focus on the potential for adjuvanted therapeutic active vaccination strategies.

## 1 INTRODUCTION

Vaccines have made an enormous contribution to the reduction of morbidity and mortality across the globe. The expression “therapeutic vaccine” may sound counter intuitive since “vaccines” are traditionally used as prophylactic medicines with the aim to prevent, rather than to treat, diseases, mostly viral, or bacterial infections. The terms “therapeutic immunization” or “therapeutic vaccines” used in this paper define vaccines, which are used therapeutically to treat a medical condition, such as an ongoing, chronic, often debilitating health problem, or an unwanted biological response. Therapeutic vaccines aim to reprogram the immune system of the patient in order to better recognize and neutralize specific deleterious molecular targets or immune cells.^1^ Hence, the vaccine could target antigens associated with an infectious (chronic) disease or non‐infectious diseases such as cancer, allergy, drug (eg, nicotine) addiction, or self‐molecules associated with autoimmune/autoinflammatory situations (eg, hypertension, neurological disorders, atherosclerosis, diabetes).^2^ A therapeutic vaccine contains a carefully chosen molecular entity, the vaccine antigen(s), which is the target of the vaccination‐induced immune response. In addition, the therapeutic vaccine generally requires a suitable adjuvant or immune modulator to induce and direct the desired type of therapeutic immune reaction. This article provides an overview of therapeutic vaccines in clinical, preclinical, and experimental use, their molecular and/or cellular targets, and the key role of adjuvants in promoting effective immune responses.

**FIGURE 1:**
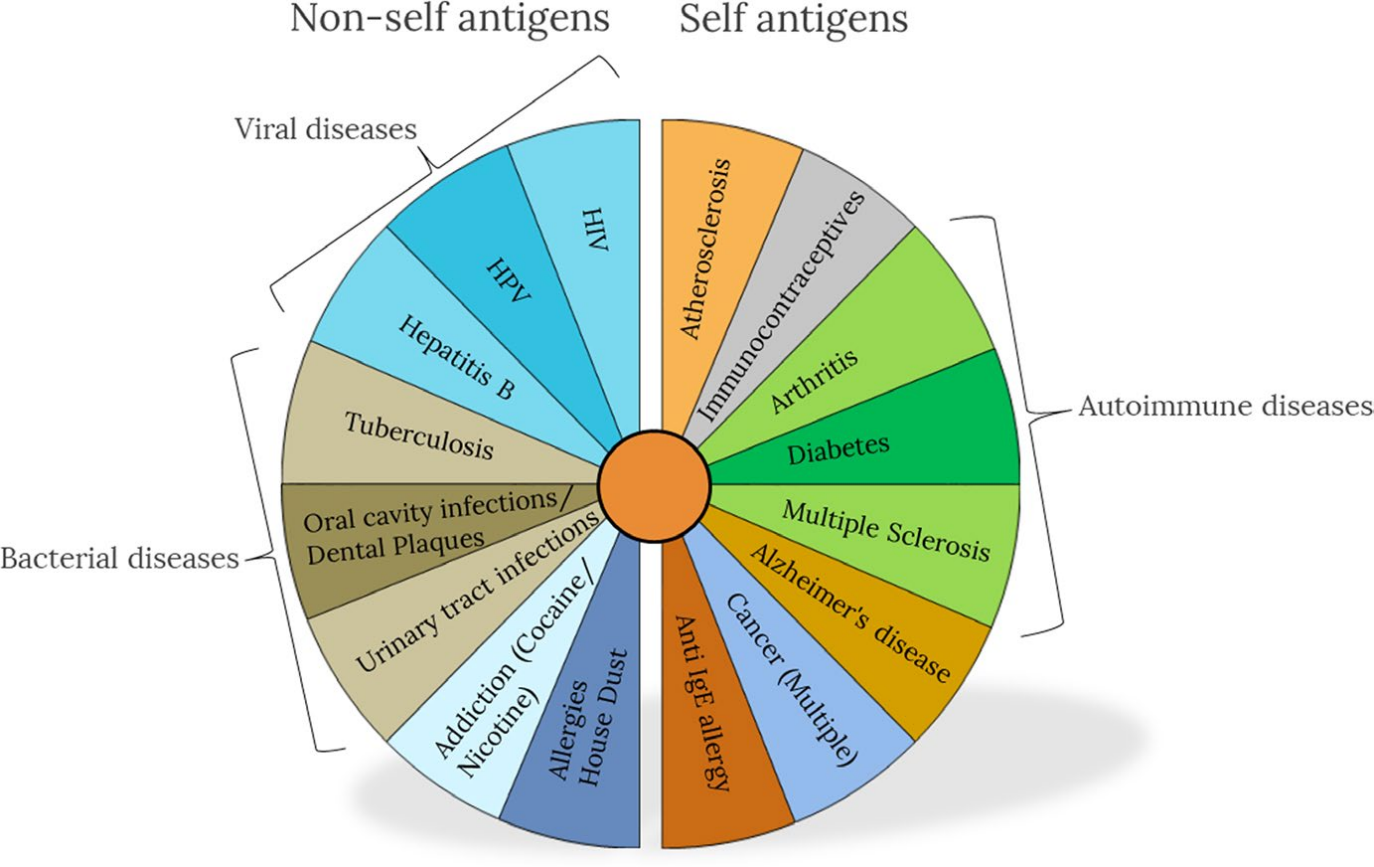
Indications for therapeutic vaccines

Given the breadth of the targeted medical conditions, therapeutic vaccines may come in many forms. Figure 1 presents an overview of the contexts where therapeutic vaccines exist or are being developed.

Since therapeutic vaccines can be designed for many indications including chronic infectious disease, allergies, addictive drugs, cancer cells, and even chronic non‐infectious disease such as atherosclerosis, a range of different vaccine approaches is required. Rational vaccine design, for both prophylactic and therapeutic vaccines, first requires elucidation of the nature of protective or beneficial immunity for a specific condition. The first step in rational therapeutic vaccine design therefore involves identification and selection of antigens, which require knowledge of the pathogenesis of the condition and accessible, and ideally stably expressed, key molecular targets. For example, an infectious target with an extracellular lifecycle (or stage) can be targeted with specific antibodies elicited by immunization, while an intracellular target (eg, a pathogen with an intracellular life cycle) needs to be targeted with a cell‐mediated immune reaction, which eventually eliminates (sacrifices) the target cell. Similarly, tumor cell targets expressed on the membrane of the tumor are accessible by antibody‐mediated cytotoxicity (ADCC), while intracellular tumor antigens need to be targeted by cellular immune responses.

The next challenge is how to generate the desired immune response against the chosen antigen, ideally with long‐term immunological memory. This requires the choice of a suitable immunostimulator that is able to induce, prolong, magnify, and steer the required immune response. This is the role for vaccine adjuvants, which with the increasing knowledge in innate immune mechanisms has moved from being “immunologists dirty little secret” to vaccinologists requisite for success. The two main objectives for the vaccine adjuvants are to (a) ensure that the antigens are delivered to those cells of the innate immune system specialized in inducing the desired immune response and (b) activate the innate immune system to direct the inducible adaptive immunity in the required direction. Modern adjuvants thus typically consist of both a delivery system, that can be composed of emulsions, liposomes, polymeric nanoparticles etc and immunostimulators that are typically ligands for so‐called pattern recognition receptors (PRRs) on the innate immune cells. These ligands are typically mimicking pathogen or damage‐associated molecular patterns (PAMPs/DAMPs), including Toll‐like receptor (TLR) agonists, C‐type lectin receptor (CLR) agonists, Nod‐like receptor (NLR) agonists, and RIG‐like receptor (RLR) agonists. Only a few adjuvants have been approved in vaccines for human use, including aluminum salts, Virosomes, MF59™, AS01™, AS03™, AS04™, and CpG, but many more are in clinical development and the repertoire will expand during the next decade. This subject has recently been reviewed in details in the book “Immunopotentiators in Modern Vaccines” which provides in‐depth insights and overviews of the most successful adjuvants, including those that have been included in licensed products and the most promising emerging technologies.^3^

Other key issues at this stage are formulation safety, stability, capacity for sterile filtration, consistency of component availability, and cost.

### 1.1 Antibody‐based immune responses against extracellular targets

Many molecules that are targets of monoclonal antibody therapies may also be candidates for actively elicited antibodies, triggered by therapeutic immunization. Hence, neutralizing antibodies induced by vaccination may be alternatives to, often very expensive, passively administered monoclonal antibodies. However, the duration of such active circulating antibody responses vs declining concentrations of passively administered monoclonals needs to be considered for safety reasons. In case of drug addictions, neutralizing antibodies induced by vaccines against cocaine or nicotine may be ideal; however, when acute reversibility or temporary inhibition of responses is the aim, passive delivery of monoclonal antibodies may be preferred over actively generated antibodies.

One of the main effector processes triggered by a vaccine is the production of antibodies by antigen‐specific B lymphocytes. Soluble antibody molecules can specifically detect and bind their antigen target, promoting its neutralization and opsonization by phagocytes (macrophages and neutrophils). Antibodies are also able to (a) block the activity of certain toxins, (b) prevent the spread of harmful infectious agents, (c) keep viruses from entering healthy cells, and (d) activate the complement cascade, which helps in microbe clearance. B cells can directly recognize antigenic moieties of extracellular pathogens through the immunoglobulins present in their outer membrane, the so‐called B cell receptors (BCR). Upon encountering the antigen, B cells become activated and differentiate into plasma cells, which produce and secrete specific IgM antibodies (without class switching) in a process known as T cell–independent activation.^4^ Activation of CD4^+^ T lymphocytes is essential for optimal B cell priming by enhancing antigen presentation.^5^ Detailed stages and processes of the germinal center and extrafollicular B cell immune response in the context of an innate immune response are recently described in detail by Shlomchik et al.^6^ T cells are activated by antigen‐presenting cells (APCs), particularly dendritic cells (DCs). Upon encountering pathogen‐associated molecular patterns (PAMPs) or danger‐associated molecular patterns (DAMPs), DCs undergo rapid maturation, modulate their surface receptors, and migrate to secondary lymphoid organs, such as lymph nodes, where B and T cell–mediated adaptive responses are initiated.^7^ In the case of extracellular pathogens and many vaccines, DCs patrolling the body can recognize and take up antigens. Depending on the nature of the antigen (bacteria, fungus, virus, etc) and immunostimulatory adjuvant moieties present, the resulting epitopes are loaded onto MHC class I or class II molecules. Epitopes derived from pathogens that enter the endosomal pathway are mainly presented by MHC class II molecules.^8^ Maturation signals induced by adjuvant molecules facilitate transport of antigen‐loaded MHC class II molecules onto the plasma membrane, where interaction with TCRs and costimulatory molecules results in activation of CD4^+^ T‐helper cells.^9^ T‐helper lymphocytes were initially subdivided into two main groups with counter‐regulatory functions depending on the cytokine pattern: a Th1 subset, which participates in cell‐mediated immunity and is associated with IFN‐γ secretion; and a Th2 subset, which was associated with enhanced proliferation of activated B cells, promoting differentiation toward plasma cells, enhanced expression of MHC class II molecules, and isotype class switching by secreting several cytokines such as IL‐4 and IL‐13.^10^ This dichotomy became outdated following the description of other CD4^+^ T‐helper populations including Th9, Th17, and Th22 cells, regulatory T cells (T_REG_), and T follicular helper (T_FH_) cells.^11^

In terms of therapeutic vaccines for treating autoimmune and inflammatory conditions and maintaining homeostasis and self‐tolerance, T_REG_ play a central role.^12^, ^13^, ^14^ T_REG_ constitute 5% of circulating T cells and are characterized by expression of the transcription factor Foxp3.^15^ In addition to these “natural” T_REG_, a population of inducible type 1 T_REG_ cells (Tr1 cells) is characterized by the secretion of IL‐10 and expression of CD49b and lymphocyte activation gene 3 protein (LAG3).^16^

**FIGURE 2:**
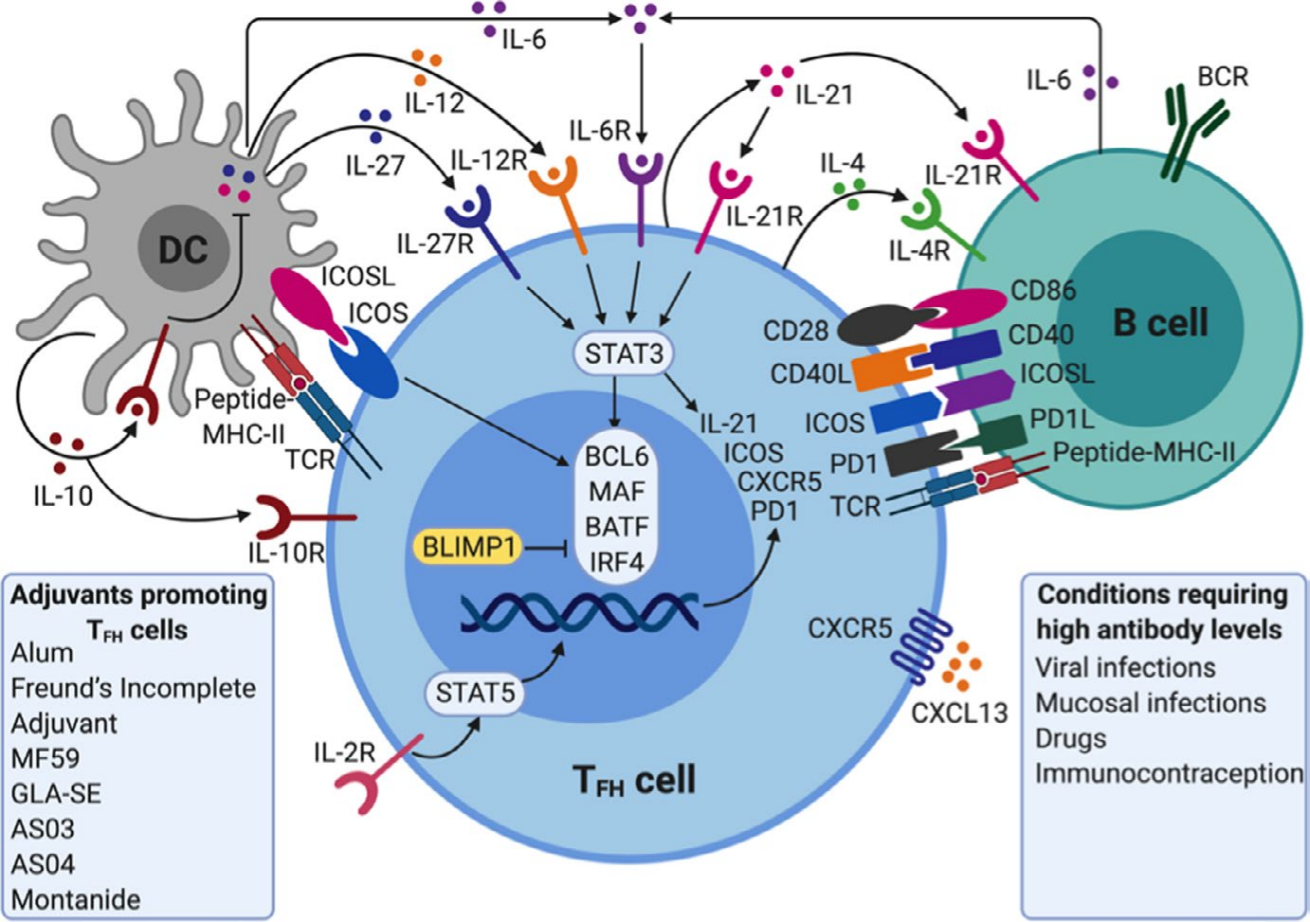
Adjuvants activate dendritic cells (DCs) to enhance generation of T_FH_ cells

Of particular relevance to vaccine‐induced antibody responses, are the follicular T_FH_ cells, which are specialized in providing T cell help to B cells.^17^, ^18^ Their specific location in the lymph node B cell follicle and cellular interactions allows them to play a key role in the induction and regulation of antibody production^19^ and B cell memory responses. This T cell–dependent B cell activation enables antibody class switching and elicits more robust responses and higher‐affinity antibodies than the T‐independent activation, highlighting the importance of optimal targeting of DCs and the subsequent instruction of CD4^+^ T cells in the adaptive response to a vaccine. These interactions and potential adjuvant interventions are highlighted in Figure 2. Differentiation of T_FH_ cells is mediated by STAT‐3 (signal transducer and activator of transcription 3), activating cytokines, secreted from DCs (IL‐6, IL‐12 and IL‐27), B cells (IL‐6 and probably IL‐27), and CD4 + T cells (IL‐21) although there are significant differences in cytokine requirements between mice and humans.^17^ The cytokines function alone or together to induce or increase the expression of transcription factors BCL 6, MAF, BATF (basic leucine zipper transcriptional factor ATF‐like), and IRF4 (interferon‐regulatory factor 4), which then causes transcription of CXCR5, ICOS (inducible T cell costimulator), IL21, and PD‐1 (programmed cell death protein 1). CD28‐CD86, CD40 ligand (CD40L)‐CD40, and ICOS‐ICOS ligand (ICOSL) interactions are central to differentiation of T_FH_ cells.

Vaccines composed of molecules with highly repetitive domains, such as polysaccharides, predominantly elicit T‐independent B cell responses, while peptide in protein‐based antigens are necessary to provide the T cell epitopes in T‐dependent B cell responses. With a view toward rational vaccine design, it is important to note that pathogen‐derived antigens often induce both pathways and these can be complementary. In addition, whereas whole pathogens possess a mixture of PAMPs that serve as adjuvants and promote DC and B cell activation, immunostimulatory adjuvants are essential to facilitate the induction of effector responses in the case of subunit vaccines.^20^ Although an increasing number of different antibody‐inducing adjuvants are included in prophylactic vaccines, including mainly aluminum salts (eg, Alhydrogel^®^, AS04™, and Adjuphos^®^) and squalene emulsions (MF59™ and AS03™), only aluminum salts are currently used in licensed therapeutic vaccines. Aluminum hydroxide is thus added to several allergy vaccines, including various pollens, animal hair/dandruff, insect bites, and house dustmites, to increase antibody responses.

## 2 THERAPEUTIC VACCINES MAINLY INDUCING ANTIBODY‐BASED IMMUNE RESPONSES

### 2.1 Allergy vaccines

Allergen immunotherapy (AIT) can provide a safe and effective treatment for allergic diseases. There are currently two types of AIT in clinical practice: subcutaneous (SCIT) and sublingual (SLIT) immunotherapy. The clinical effect of both SCIT and SLIT has been well demonstrated in several randomized, double‐blind, placebo‐controlled clinical trials, showing significant reductions in symptoms and medication scores.^21^ The principle behind AIT is to expose the allergic individual to the specific allergen(s), leading to immunological tolerance or altered antibody responses with a lasting effect, even after termination of the treatment.^22^, ^23^

Adjuvants and adjunct therapies have, in the context of AIT, been investigated since the 1990s, with the aim to either increase the efficacy, lower the adverse events, or shorten the treatment. So far, we have not seen a major breakthrough, but several clinical trials are still aiming to change this picture (Table 1). As previously mentioned, aluminum hydroxide (alum) has been the adjuvant of choice in SCIT. The effect of alum can partly be attributed to its adsorptive properties, which leads to increased immunogenicity due to the slow and sustained allergen release from the alum particles ^24^ (depot effect). Importantly, allergens strongly adsorbed to alum particles are less accessible to the immune system, which has been shown to decrease local side effects.^25^ A number of pathways have been implicated in alum adjuvanticity, including DNA release,^26^ prostaglandin E2, and cholesterol interaction.^27^ The mineral salt, calcium phosphate, and the natural amino acid L‐tyrosine are also used as depot adjuvants in SCIT. Both of these adjuvants enhance IgG production with a limited increase of IgE.^28^

**TABLE 1:** Adjuvants used in clinical trials against allergy

Adjuvant	Delivery system	Immunostimulator	Receptor	Disease	NCT number
Al(OH)_3_	‐	Multiple theories	‐	Grass pollen allergy	01538979
MPLA	‐	MPLA	TLR4	Tree pollen allergy	00118625
GLA	‐	TLR4 agonist	TLR4	peanut	03463135

Note

The table displays adjuvants, which have been or are currently used in clinical trials of allergy. Clinical trials in which the adjuvants have been used are identified by the Clinical Trials gov. identifier number (NCT). Numbers are examples, and only one number is given although some adjuvants are used in several clinical trials.

More recently, Toll‐like receptor (TLR) ligands have been applied as adjuvants in SCIT. One example is monophosphoryl lipid A (MPL), which is a non‐toxic derivative of lipopolysaccharide (LPS) which interacts with TLR4 on innate immune cells and can promote Th1 responses.^29^ In combination with L‐tyrosine, MPL is being investigated in phase III clinical studies as an adjuvant in short‐course SCIT treatment of pollen‐induced allergies. However, the most recent of these studies, treating birch pollen allergics, failed to meet the primary endpoint of reducing the combined symptom and medication score (Study B301). Glucopyranosyl lipid A (GLA) is similarly a TLR4 ligand, inducing a Th1 response by activating dendritic cells.^30^ This adjuvant is currently being evaluated in a clinical study with SLIT treatment of peanut allergic patients (NCT03463135). Attempts have been made to improve AIT using adjuvants targeting TLRs other than TLR4. CpG oligodeoxynucleotides (CpG‐ODN) resemble bacterial DNA and bind to the endosomal receptor TLR9. In animal models of asthma, it has been shown that CpG‐ODN downregulate the allergic Th2 response and induce a Th1 and T‐regulatory response.^31^ This concept was tested by SCIT treatment of ragweed allergic patients where the antigen Amb a 1 was conjugated to a CpG‐ODN. Initial results from a phase II study showed promising results, but in a later phase IIb study, the effect was not different from placebo.^28^

Nanoscale adjuvants, including virus‐like particles (VLP), have also been tested for efficacy in SCIT. VLPs are virus‐shaped particles made of coat proteins or capsids from viruses or bacteriophages. These particles are highly immunogenic and linking allergens to their surface enhances their immunogenicity. However, it has been demonstrated that VLP bound allergen has a strongly impaired ability to bind to surface bound IgE and to induce mast cell degranulation.^32^ The enhanced immunogenicity was demonstrated in a small clinical trial, where high titers of specific antibodies were observed after immunization with the antigen Der p 1 conjugated to VLP.^33^ Furthermore, VLP combined with CpG‐ODN has been tested as a SCIT adjuvant in clinical trials, where it showed reduction of clinical symptoms in house‐dust mite allergic patients.^34^ Interestingly, this reduction could be seen both with and without coupling of allergen to the VLPs.^35^

An alternative to adjuvants is co‐administration of adjunct biopharmaceuticals. In several studies, AIT has been combined with omalizumab (anti‐IgE antibody) for the treatment of allergic rhinitis or asthma, resulting in fewer side effects compared to AIT alone.^36^ In food allergy, it has been shown that addition of omalizumab to oral immunotherapy was beneficial effect in terms of adverse events, but had no effect on tolerance induction or sustained responses.^36^ Dupilumab, an anti‐IL‐4 receptor alpha‐specific antibody, is currently being investigated as adjunct therapy to peanut AIT. Mouse studies have indicated that peanut AIT and anti‐IL‐4R antibody treatment show a synergistic treatment effect.

### 2.2 Vaccines for substance use disorders

Addictive drugs are a chemically heterogeneous group of small molecules that are able to pass the blood‐brain barrier (BBB) and target the distinct neurotransmitter systems in the brain. Vaccines have the potential to induce anti‐drug antibodies that cannot cross the BBB but can potentially bind to the drug and inhibit its transport to the brain, without altering brain function. Because the addictive drugs are small non‐immunogenic molecules, a hapten‐carrier approach is used which requires conjugation to a carrier protein to enhance the induction of drug‐specific antibodies. For example, the key components of a conjugate nicotine vaccine are: a B cell epitope, in this case nicotine; a T cell epitope, provided by the carrier protein as used in polysaccharide conjugate vaccines for capsulated bacterial pathogens and an adjuvant that enhances vaccine immunogenicity.^37^ Although the vaccine principle is the same for all drugs, when designing the hapten vaccine, it is important to consider drug properties such as size, chemical structure, metabolism, and biodistribution.^38^ The efficacy of induced antibodies will depend on a number of variables including the carrier, hapten density, aggregates and adducts, and a choice of adjuvant.^38^, ^39^, ^40^, ^41^, ^42^, ^43^

Nicotine, a psychostimulant, is the main addictive component in tobacco products. Nicotine‐targeting vaccines have been the best studied of all addictive drug vaccines, with six candidates reaching clinical trials.^44^ NicVax is the first‐generation anti‐nicotine vaccine that has demonstrated promising results in phase 2 trials, but has failed to show improvement in smoking cessation compared to placebo in phase III trials.^45^, ^46^, ^47^ Esterlis et al^48^ have shown that the vaccine resulted in only a 12.5% decrease of nicotine concentration in the brain. NicVAX consists of a hapten, 3’aminomethylnicotine, conjugated to the exoprotein A from *Pseudomonas aeruginosa* and alum as an adjuvant.^44^

Improved responses have been seen with new hapten designs involving conjugation to cross‐reactive material 197 (CRM197), a non‐toxic derivative of diphtheria toxin (DT), and addition of CpG adjuvant (TLR agonist) in addition to alum.^41^, ^49^, ^50^, ^51^ Indeed, this formulation induced higher titers of nicotine‐binding antibodies in rats and non‐human primates (NHPs), and the study showed that a combination of alum and CpG adjuvants can enhance both the antibody titer and affinity.^49^ Because of the positive preclinical results, the vaccine (NIC7‐001) is currently being tested in a phase I clinical study; however, the results are not yet available. The N4N vaccine is another second‐generation vaccine that has shown promise for nicotine vaccination.^52^ The N4N hapten is a covalent modification of pyridine and has much higher nicotine affinity than 3’aminomethylnicotine from the NicVax vaccine. The N4N hapten is conjugated to flagellin but has not yet been tested clinically.

A different vaccine approach for inducing drug‐specific antibody responses involves particle‐based vaccines, which are built from either polymers, liposomes, peptides, virus‐like particles, or other combinations.^53^, ^54^, ^55^ These self‐assembling particle vaccines are anticipated to enhance the activation of antigen‐presenting cells (APC), to promote stronger T‐helper cell responses, and to stimulate the differentiation of memory B cells.^56^, ^57^ Additionally, the hapten load can be controlled and the delivery of adjuvants and other immunomodulators to APCs made more efficient.^42^ The nanoparticle‐based vaccine SEL‐068 from Selecta Bioscience consists of nicotine bound to the surface of polymers, a synthetic TLR ligand, and a T‐cell helper peptide. In preclinical studies in non‐human primates, the vaccine blocked the development of nicotine discrimination, a behavioral experimental procedure to test the effect of nicotine.^58^ The Selecta group showed that codelivery of an antigen with a TLR7/8 or TLR9 agonist in synthetic polymer nanoparticles increased drug immunogenicity with minimal systemic production of inflammatory cytokines.^59^ SEL‐068 is currently being evaluated in phase 1 clinical trials.

Another particle‐like vaccine in preclinical studies incorporates a synthesized short trimeric coiled‐coil peptide (TCC) that creates a series of B and T cell epitopes with uniform stoichiometry and high density.^60^ Vaccination with this antigen and alum and a TLR4 agonist (GLA‐SE) could prevent 90% of a nicotine dose equivalent to three smoked cigarettes from reaching the brain. The TLR4‐based adjuvant, as a potent stimulator of T cell–mediated antibody responses, has shown superiority compared to alum, with higher antibody titers and improved antibody affinities.

More recently, a hybrid nanoparticle‐based nicotine vaccine (NanoNiccine) has been developed with an aim to improve specificity and induce more sustained responses.^61^ NanoNiccine is composed of a poly(lactide‐co‐glycolide) acid (PLGA) core, keyhole limpet hemocyanin (KLH) as an adjuvant protein enclosed within the PLGA core, a lipid layer, and nicotine haptens conjugated to the outer surface of the lipid layer. The vaccine showed superior immunogenicity compared to traditional nicotine‐protein conjugate vaccines. The particles were efficiently taken up by dendritic cells, and the principal adaptive immune response detected was the induction of antigen‐specific IgG antibodies. The same group have demonstrated that the immunogenicity of the NanoNiccine vaccine can be further improved by modulating factors such as particle size,^62^, ^63^ hapten localization, and density,^62^, ^63^ combinations of adjuvants,^64^, ^65^ conjugation of potent carrier proteins,^64^, ^65^ and degree of pegylation.^66^ In addition to the great potential of novel adjuvant approaches to enhance the magnitude and quality of the anti‐drug antibody response, the nature of the hapten used and the degree to which it can trigger high affinity anti‐drug antibodies is pivotal. However, detailed discussion of this topic is outside the scope of this review.

### 2.3 Vaccines against chemical hazards

DDT (1,1,1‐trichloro‐2,2‐bis (p‐chlorophenyl) ethane) has been used widely as a pest control agent but was banned in 1970 because of its harmful effects on wildlife and human health.^67^ This compound is therefore a chemical hazard for both human health and the environment. The biodegradation of DDT is very slow,^68^ and in animals and humans, the DDT accumulates and is stored in adipose tissue.^69^ When an organism is a part of the food chain, a biomagnification occurs, where the hazard is accumulated.^70^ Although the liver is able to transform some of the DDT to DDE or DDD,^71^ these degradation compounds are not eliminated, but are stored even more avidly.^72^

Research has been conducted into the potential of the immune system to eliminate DDT following vaccination. The toxin DDT was made immunogenic by conjugating it with keyhole limpet hemocyanine (DDT‐KLH). Mice were immunized subcutaneously using aluminum hydroxide adsorbed DDT‐KLH conjugate, where the second group only received KLH adsorbed to aluminum hydroxide, and the third group was used as a control to provide information on DDT levels in serum in untreated animals. The mice were then fed with chow containing 40 mg/kg of DDT for 45 days.^73^ The concentration of DDT and its metabolites (DDE and DDD) was analyzed in various tissues, and DDT‐specific antibody titers were determined. Higher antibody responses were detected in mice vaccinated with the DDT‐KLH conjugate, and DDT, DDE, and DDD levels in adipose tissue, blood, brain, and spleen were significantly reduced, compared to the group that received native unconjugated DDT.^73^ This demonstrates that immunization against a chemical hazard may be used to treat animals or humans exposed to high levels of such toxic compounds, as well as prevent them from having these compounds accumulating in their body.

### 2.4 Alzheimer's disease vaccines

Alzheimer's disease (AD) is a progressive neurodegenerative disorder, characterized by plaques of misfolded amyloid β (Aβ) and tau protein aggregates in neural tissue, and cerebrovascular dysfunction resulting from damaged small blood vessels in the brain,^74^ which eventually leads to dementia in the elderly population. Currently, it is considered an incurable disorder with limited treatment options. The mechanism(s) underlying the cognitive decline in Alzheimer's disease still has not been clearly unraveled, which makes it difficult to judge whether current therapies target the symptoms, rather than the key molecule(s) causing the pathology. In AD transgenic animal models, immunotherapeutic targeting of B cell epitopes of (misfolded) Aβ and/or tau seemed a most logical strategy to target neural plaques or vascular aggregates. Indeed, both vaccines, and passive antibody‐based interventions, resulted in a clear reduction of Aβ pathology and cognitive benefits, with limited local inflammatory adverse effects. However, despite these promising preclinical results, such approaches in human studies showed limited evidence of significant clinical benefits, even despite the postmortem observed clearance of amyloid pathology, and acceptable tolerability.^75^, ^76^, ^77^

Several redesigned vaccines against the (modified) variants of soluble or aggregated Aβ and tau protein (with or without protein carrier Qβ or KLH, lacking common T cell epitopes) formulated with saponin QS‐21, liposomes plus TLR4 agonist, or KLH‐alum, as immune modulating adjuvants, are being evaluated in early‐stage trials.

### 2.5 Atherosclerosis vaccines

Accumulation of leukocytes in the intima of the arterial wall and subsequent uptake of lipids in the form of low‐density lipoprotein (LDL) by macrophages initiate the formation of atherosclerotic lesions in large and medium sized arteries. As lesions grow, they become increasingly unstable, ultimately causing them to rupture, which can result in thrombotic occlusion of blood vessels. As such, atherosclerosis is the major underlying pathology of cardiovascular events like myocardial infarction or stroke and is the leading cause of death in the Western world. Although current treatment strategies focus on controlling LDL levels (eg, by statins, PCSK9 inhibitors), immunomodulatory interventions, including vaccination, are currently in preclinical and clinical investigation.

Development of atherosclerotic plaques is driven by an inability to clear LDL from the lesion, resulting in a chronic inflammatory reaction involving both innate and adaptive immunity, and therefore, LDL has been the most intensively studied antigen. Indeed, LDL‐specific T cells have been identified in atherosclerotic lesions^78^ and circulating auto‐antibodies against LDL or modified LDL can be readily detected in most individuals.^79^ Interestingly, induction of anti‐LDL antibodies by immunizing rabbits with chemically modified LDL particles suspended in the water‐in‐oil emulsion, Complete Freund's adjuvant (CFA), or AdjuPrime had a protective effect with less prominent atherosclerotic lesions in the vaccinated animals.^80^, ^81^ Similarly, vaccination with peptides derived from the largest protein in LDL, ApoB100, formulated with aluminum phosphate and cationized BSA, or CFA, reduced atherosclerosis in mice.^82^, ^83^

### 2.6 Cancer vaccines against tumor‐associated antigens (TAAs)

**FIGURE 3:**
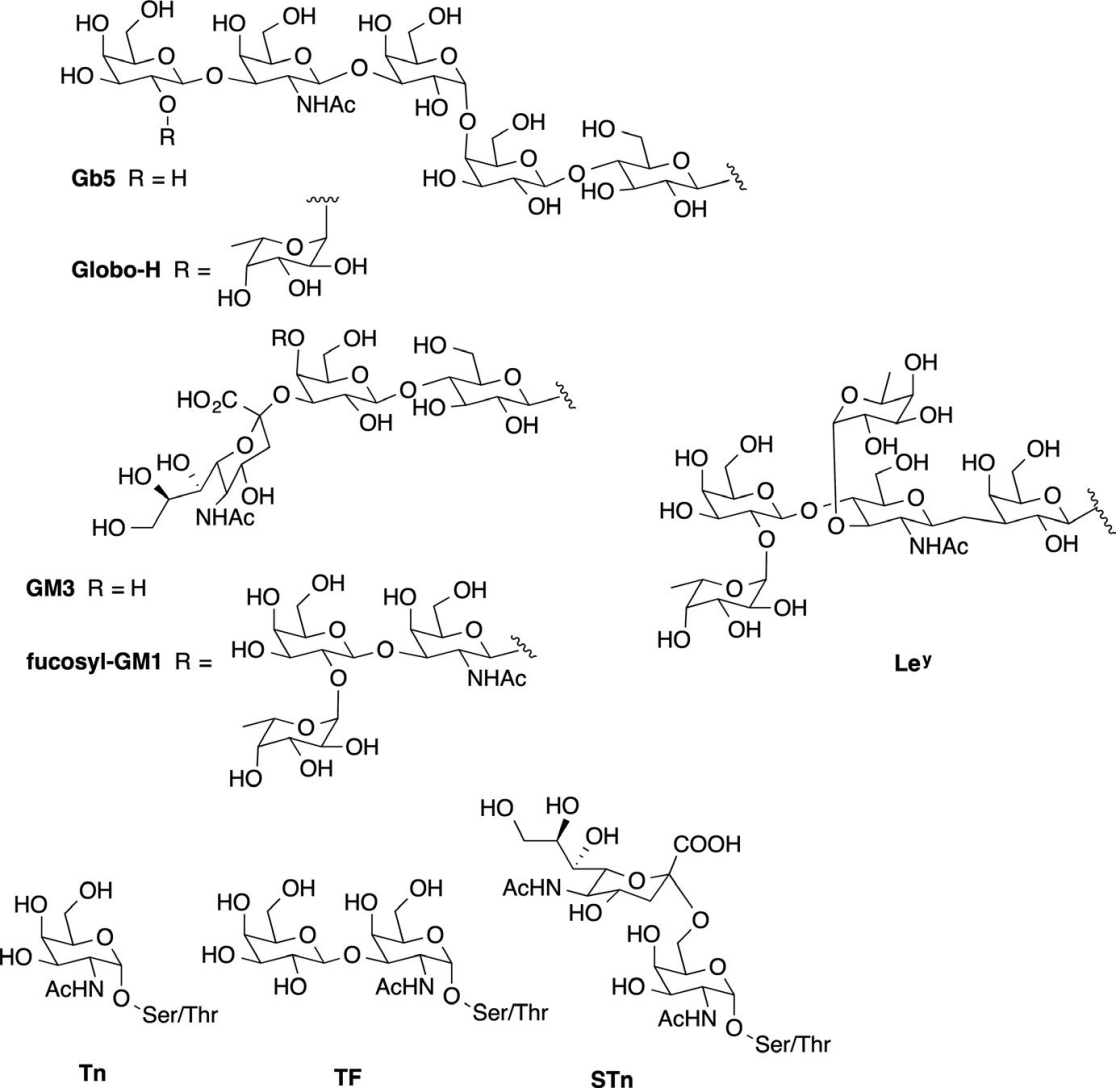
Examples of major Tumor‐associated carbohydrate antigens (TACAs)

Antibodies targeting tumor‐associated cell surface antigens have been shown to be able to eliminate circulating tumor cells,^84^ and vaccine‐induced antibodies against these antigens, especially carbohydrates, have correlated with improved prognosis in clinical settings.^85^ Thus, tumor‐associated carbohydrate antigens, (TACAs), which are aberrantly expressed at the surface of tumor cells compared with normal cells, either as glycolipids (GM2, GD2, GD3, Globo H) or as mucin glycoproteins (Tn, TF, STn, MUC1),^86^ have become important targets for antibody recognition and immune attack against cancers (Figure 3, Table 2). A relationship exists between tumor type and expression of these abnormal TACAs.^87^ Since the first pioneering hypotheses on the cell biological significance of aberrant glycosylation appeared in 1985,^88^ efforts focused on the development of anti‐TACA‐based cancer vaccines have expanded.^85^, ^89^, ^90^, ^91^ Despite these efforts, however, no anti‐TACA cancer vaccine has been approved by the FDA. The failures experienced by the TACA‐based vaccines that entered clinical trials can be ascribed to several factors, being the most compelling one that carbohydrate antigens are strictly B cell epitopes and induce a T cell–independent humoral response. Moreover, it should be considered that targeting TAAs with antibody‐based immune responses may not target variant tumor cells expressing different TAAs in case of tumor heterogeneity, resulting in tumor immune escape from vaccine‐induced immune reactions.^92^

**TABLE 2:** Antigens targeted in therapeutic antibody‐inducing cancer vaccines

Antigens	Tumors
GM2, globo H, sTn, TF, Le^y^	Ovary
GM2, Tn, sTn, TF, Le^y^	Prostate
GM2, globo H, Tn, sTn, TF, Le^y^	Breast
GM2, fucosyl GM1, polysialic acid, globo H, sialyl Le^a^	Small‐cell lung cancer
GM2, GD2, GD3	Sarcoma
GM2, GD2, GD3, polysialic acid	Neuroblastoma
GM2, GD2, GD3	Melanoma

Vaccine‐elicited antibodies against TAAs can mediate elimination of tumor cells by complement‐dependent cytotoxicity (CDC) and/or antibody‐dependent cellular cytotoxicity (ADCC). Another important function of these antibodies is opsonization of tumor cells to promote their uptake by antigen‐presenting cells. The basis for vaccines including this type of TAA is the correlation between overall antibody titer against tumor cells and the CDC or ADCC effector mechanisms. Antibody‐inducing vaccines in mice and cancer patients have included cell surface carbohydrate antigens conjugated to carrier proteins such as KLH or bovine serum albumin (BSA), co‐administered with a variety of distinct types of adjuvant, for example the natural saponin adjuvant QS‐21, the semisynthetic saponin adjuvant mixture GPI‐0100, β‐glucan, or the nanoparticulate product GlycoVaxGM_3_ with Montanide ISA 51 (Table 3).^93^ Some other glycoprotein antigens such as MUC1 and KSA in epithelial cancers, PSMA in prostate cancers, and MUC16 in ovarian cancer are also abundantly expressed on other types of cancers and have also been a target for antibody‐inducing vaccines.

**TABLE 3:** Adjuvants used in cancer vaccines

Adjuvant	Delivery system	Immuno‐stimulator	Receptor	Type of cancer	Antigen^a^	NCT number^b^
Montanide ISA51	W/O emulsion	‐	‐	1.CNS tumors 2.Breast 3.Leukemia	1.TAA peptide mix 2.P10s‐PADRE 3.PR1 peptide	1.00935545 2.01390064 3.00004918
Hiltonol	‐	Poly:IC‐LC	TLR3/ RIG‐I	1.Solid tumors 2.Lung 3.Melanoma	1.Neo‐antigen 2.MUC‐1 3.pBCAR3 peptide	1.02721043 2.01720836 3.01846143
AS15	Neutral liposomes	CpG/MPLA/QS‐21	TLR9/4	1.Melanoma 2.Breast 3.Lung	1.MAGE‐A3 2,MAG‐Tn3 3.PRAME	1.01425749 2.02364492 3.01853878
CAF09b	Cationic liposomes	MMG/ Poly:IC	Mincle/ TLR3/RIG‐I	1.Solid tumors 2.Prostate	1.Neo‐antigen 2.Bcl‐XL	1.03715985 2.03412786
ISA51 + Hiltonol	W/O emulsion	Poly:IC‐LC	TLR3/ RIG‐I	1.Melanoma 2.AML 3.Glioma	1.NY‐ESO‐1 2.WT1 peptide 3.GAA/TT‐peptide	1.01079741 2.01842139 3.00795457
ISA51 + CpG7909	W/O emulsion	CpG	TLR9	1:Lung 2:esophageal 3:Melonma	1:NY‐ESO‐1b 2:URLC10‐177 3: MAGE‐3.1	1:00199836 2:00669292 3:00085189
ISA51 + Aldara	W/O emulsion	Imiquimod	TLR7/8	1:Melanoma 2:Prostate	1:gp100 2:Pros.spec. peptide	1:00273910 2:02452307
Detox B	Oil droplet emulsion	MPL/mb. cell wall	TLR4+?	1:Solid tumors 2:Breast	1:ras peptide 2:THERATOPE	1:00019006 2:00003638
QS‐21	‐	QS‐21	Unknown	1:Ovarian 2:Breast 3:Prostate	1:Globo H‐GM2 2:sialyl Lewisª 3:MUC‐2‐KLH	1:01248273 2:00470574 3:00004929
Iscomatrix	ISCOMs	QuilA	Unknown	Melanoma	NY‐ESO‐1	00199901
Resiquimod	‐	Resiquimod	TLR7/8	1:Melanoma 2:Bladder 3:Glioma	1:CDX‐1401 2:CDX‐1307 3:Tumor lysate	1:00948961 2:01094496 3:01204684
Resiquimod + Hiltonol	‐	Resiquimod/ Poly:IC‐LC	TLR7/8/ TLR3/RIG‐I	Melanoma	CDX‐1401	00948961
Sargramostim	‐	GM‐CSF	GM‐CSFR	1:Breast 2:Lung 3:Ovarian	1:HER2 Peptide 2: ras peptide 3:ALVAC‐NY‐ESO‐1	1:00003002 2:00005630 3:00803569
GLA‐SE	‐	GLA	TLR4	1:Melanoma 2:Ovarian 3:Lung	1:MART‐1 2:IDC‐G305 3:CMB305	1:02320305 2:02015416 3:02387125
ISA51 + GM‐CSF	W/O emulsion	GM‐CSF	GM‐CSFR	1:Multiple Mye. 2:Leukemia 3:Melanoma	1:SVN53‐67‐KLH 2:PR1 peptide 3:MART‐1	1:02334865 2:00004918 3:00031733
Alhydrogel	‐	‐	‐	Prostate cancer	rsPSMA	00705835
Allostatine	‐	?	?	Various	‐	‐

Note

The table displays an overview of adjuvants, which have been or are currently used in clinical trials of cancer vaccines. The type of cancer, antigen type, and Clinical Trials gov. identifier number (NCT) is given.

AML, Acute Myeloid Leukemia.

^a^ For therapeutic vaccines containing multiple antigens, only one is displayed in the table.

^b^ Note that although some adjuvants are used in trials against several cancer types, only up to three trial (NCT) numbers are displayed.

Despite some promise, the clinical anti‐tumor effects of the antibodies induced by early formulations of carbohydrate‐protein conjugate vaccines were not significant, likely due to the advanced stages of disease and tumor immune escape.^94^ These disappointing outcomes in terms of overall survival have driven the development of alternative synthetic carbohydrate vaccines, which have been shown to be immunogenic without the need for a conjugated protein carrier or an external adjuvant. Some of these new vaccine constructs have focused on glycosylated MUC1 B‐cell epitopes, which have elicited robust IgG antibody responses in MUC1.Tg mice that were able to kill MUC1‐bearing tumor cells by CDC and ADCC.^95^, ^96^ The antibodies showed selectivity toward human breast cancer tissues, and the vaccine candidates exhibited therapeutic anti‐tumor effects, paving the way for potential translation into the clinic.

In recent times, the development of TACA‐based vaccines has placed special emphasis on the impact of several factors on the immune response: that is, (a) antigen modification and density, (b) the carrier, and (c) the selected adjuvant. Different strategies have thus been proposed to induce improved anti‐TACA immune responses, spanning from synthetic modification of antigens to multiantigen vaccine constructs, relying on nanosized, peptidic, or protein carriers.^91^ In Box 1, we summarize a number of cancer vaccine prototypes where the objective is targeting of TACAs with antibodies.

BOX 1 **Cancer vaccine prototypes based on TACAs targeted by antibody induction**

**Le^y^ AND SLe^a^‐BASED VACCINES**

A Le^y^‐based vaccine, effective in preclinical trails, was generated by conjugating the synthetic pentasaccharide Le^y^ to KLH and injected with QS‐21 as an adjuvant. Vaccination triggered IgM and, to a lesser extent, IgG antibodies and proved to be selectively toxic to Le^y^‐positive cells.^97^ This potential vaccine entered a phase I clinical trial against ovarian cancer, but was discontinued because the antibodies induced were low affinity IgM molecules. The hexasaccharide SLe^a^ (CA19‐9) was also used as a TACA linked to KLH and co‐administered with QS‐21 or with the semisynthetic saponin adjuvant mixture GPI‐0100. In animal tests, vaccination with SLe^a^‐KLH alone induced moderate antibody titers, which were increased by using GPI‐0100 as an adjuvant. The induced IgM and IgG antibodies mediated cytotoxicity against the SLe^a^‐positive human adenocarcinoma cell line SW626. No cross‐reactivity to other or similar carbohydrate antigens (SLe^x^, Le^a^ or Le^y^) was detected.^98^

**GLOBO H‐BASED VACCINES**

The first total synthesis of the Globo H hexasaccharide was reported in 1995.^99^ The enzymatic synthesis of Globo H by large scale manufacturing of this demanding TACA enabled the development of Globo H‐based vaccines. A number of different carriers and adjuvants have been tested to enhance Globo H‐specific immune responses. Vaccination with the resulting Globo H‐KLH conjugate in combination with QS‐21 in a phase I clinical trial for breast cancer was well‐tolerated, and increased CDC and ADCC activity was observed in several patients. In a second‐generation vaccine in which Globo H was linked to the diphtheria toxin mutant CRM_197_ and used with the glycolipid adjuvant C34, a synthetic ligand for CD1d, improved immune responses were obtained.^100^

**GD_2_‐, GD_3_‐, GM_3_‐, AND FUC‐GM_1_‐BASED VACCINES**

GD_2_ and GD_3_ are neuroblastoma TACAs that were used in a vaccine that entered a phase I clinical trial.^101^ The two disialogangliosides, extracted from natural sources (rabbit brain and bovine buttermilk, respectively), were linked to KLH and administered with the QS‐21‐related saponin OPT‐821 or β‐glucan as adjuvants. The bivalent GD_2_/GD_3_ antigen/β‐glucan vaccine induced anti‐GD_2_ and anti‐GD_3_ antibody responses and was well tolerated, without major toxicity signs or induction of neuropathic pain.

The monosialoganglioside GM_3_ has also been considered as a TACA. However, a lactone metabolite of GM_3_ has been demonstrated to be a more discriminating antigen on cancer cells. Under physiological conditions, the expression of this lactone is below the recognition threshold.^102^ Thus, GM_3_ has been the subject of extensive synthetic studies, and structural analogues have been proposed for the development of cancer vaccines.^103^, ^104^, ^105^

The NeuGcGM_3_ ganglioside is a natural, GM_3_‐containing glycoantigen overexpressed on melanoma cells and found in other carcinomas, but poorly expressed in most normal human tissues. NeuGCGM_3_ was included in a proteoliposome of *Neisseria meningitidis* and was tested as a nanoparticulate product (GlycoVaxGM_3_) with Montanide ISA 51 as an adjuvant. Patients with melanoma^106^ or breast cancer^107^ treated with GlycoVaxGM_3_ developed IgM and IgG antibodies to NeuGcGM_3_ that could mediate complement‐dependent cytotoxicity (CDC) against P3X63 myeloma cell lines. Clinical trials with GlycoVaxGM3 are ongoing.^108^

The fucosyl‐GM_1_ hexasaccharide is expressed on many small‐cell lung cancers but not on normal cells. The synthetic fucosyl‐GM_1_ linked to KLH was employed in combination with QS‐21 as an adjuvant.^109^ Upon vaccination, an IgM antibody response against fucosyl‐GM_1_ and against tumor cells expressing fucosyl‐GM_1_ was elicited.

**Tn‐, STn‐, TF‐, AND STF‐BASED VACCINES**

The MUC1‐related Tn, TF, STn, and STF TACAs, expressed in more than 90% of primary adenocarcinomas, are considered pancarcinoma antigens and have been widely used to design cancer vaccines. They can be found attached through *O*‐glycosidic linkages to serine or threonine residues of tandem repeat peptide sequences. A synthetic, Tn/TF‐containing MUC1 peptide sequence conjugated to bovine serum albumin (BSA) has been developed as a vaccine candidate. A higher antibody response was obtained when a Tn or a TF residue was linked to a threonine residue (19). The same authors also synthesized the MUC1 tandem peptide glycosylated with STn and 2,6‐STF antigens at a serine residue (S15), maintaining Tn or TF antigens linked to threonine (T9). After conjugation to BSA, the vaccine candidates were co‐administered with complete Freund's adjuvant for the first inoculation and with incomplete Freund's adjuvant for each subsequent immunization.^110^ These vaccines generated a strong antibody response, with elicitation of IgM antibodies and IgG1 as the predominant antibody isotype.

A tricomponent vaccine comprising the TLR2 ligand Pam_3_CysSK_4_ as an adjuvant, a T‐helper cell epitope peptide, and a Tn glycosylated MUC1‐derived peptide induced a potent humoral and cellular response, including CTL and ADCC‐mediating antibodies. This vaccine was able to generate a therapeutic response due to specific immunity against MUC1 and to a non‐specific anti‐tumor response elicited by the adjuvant.^95^

Another example of self‐adjuvating potential cancer vaccine was developed by covalent conjugation of α‐galactosylceramide (αGalCer), a CD1d and invariant natural killer T (*i*NKT) cell ligand, with sialyl Tn (STn) TACA. This vaccine showed remarkable efficacy in inducing a strong STn‐specific IgG response.^111^

In an effort to improve the multivalent presentation of TACAs as well as to improve anti‐glycan immunity, the formulation of Tn antigen conjugated to αGalCer into liposomes was recently published.^112^ Liposomes containing 1,2‐diasteraroyl‐*sn*‐glycero‐3‐phoshpocholine (DSPC) and cholesterol were prepared by lipid extrusion and co‐formulated with Tn‐αGalCer glycoconjugate and αGalCer. The combination of DSPC and cholesterol for liposomal vaccines was known to induce a strong antibody (particularly IgG1) response in mice.^113^

In another vaccine design, the Tn antigen was linked to virus‐like particles (VLP) of the bacteriophage Qβ, eliciting higher and more diverse antibody responses.^114^ A four‐component self‐adjuvating multivalent cancer vaccine prototype was also proposed, relying on a cyclic peptide scaffold presenting four residues of Tn antigen and decorated with a B‐cell epitope. The multivalent scaffold also incorporated the peptidic Th epitope (PADRE), a CTL epitope from OVA, and the TLR2 ligand palmitic acid as an adjuvant. In mouse models, vaccination with this construct elicited IgG antibodies and offered protection against tumor growth.^115^ A STn‐KLH glycoconjugate vaccine, THERATOPE^®^, reached phase III clinical trials, although with a poor clinical outcome. In the pilot study, the STn‐KLH vaccine was administered to patients with metastatic breast cancer pretreated with cyclophosphamide. All patients developed IgM and IgG antibodies that recognized both the synthetic and natural STn disaccharide.^116^

Given the ability of certain zwitterionic polysaccharides (ZPSs) to evoke an MHC class‐II‐mediated T‐cell response, a new protein‐free, saccharide‐based vaccine construct was proposed consisting of Tn antigen chemically conjugated to PSA1 ZPS from *Bacteroides fragilis*.^117^ This Tn‐PSA1 vaccine was administered to C57BL/6 mice in combination with TiterMaxGold as an adjuvant, generating high antibody titers specific to the Tn moiety. Subsequently, the same authors developed a related STn‐PSA1 vaccine candidate. In mice co‐administered with MPLA as an adjuvant, this vaccine elicited strong and functional humoral and cellular responses, with high titers of antibodies able to bind to and mediate CDC against STn‐expressing cancer cell lines.^118^

Currently, the most intensively studied TACAs for the development of cancer vaccines are Lewis determinants (Le^a^, Le^y^, SLe^a^) and glycans of the Globo class (Gb5, Globo H), which are overexpressed in a range of tumors, as well as carbohydrates present in gangliosides (GM_2_, GM_3_, GD_2_, fucosyl GM_1_) overexpressed in melanomas, lung, colon, prostate cancer, and mucin‐related TACAs (Tn TF, STn, STF) characterized by their rather simple structures and largely expressed in adenocarcinomas. Expression of these TACAs correlates with tumor malignancy.

## 3 THERAPEUTIC VACCINES MAINLY INDUCING ENHANCED OR MODIFIED CELLULAR IMMUNE RESPONSES

### 3.1 Cell‐based immune responses to intracellular targets

Cytotoxic CD8^+^ T lymphocytes (CTLs) play a crucial role in tumor control and chronic infections by intracellular pathogens. Indeed, they display a large spectrum of cytotoxic mediators in response to the specific recognition of tumor antigens presented in the context of the MHC class I complex on the surface of the target cell. CTLs mediate tumor or virus‐infected cell death by direct or indirect processes: (a) the release of lytic molecules, (b) the binding to pro‐apoptotic receptors expressed by tumor cells, (c) the secondary recruitment of effector cells, and (d) the increase of tumor cell recognition via the induction of MHC‐I molecule expression. Such highly cytotoxic capacities make their induction and infiltration into the tumor an attractive therapeutic strategy.

### 3.2 Therapeutic cancer vaccines

Anti‐tumor therapeutic vaccination should generate not only tumor‐specific CD8^+^ T cells but also tumor‐specific tissue‐resident memory T cells (Trm) at the tumor site.^119^ Indeed, Trm are emerging as an essential actor in tissue immunosurveillance due to their specific expression of defined adhesion molecules (CD103 or CD49a integrins, chemokine receptors) facilitating their retention in tissues and consequently into the tumor. Their well‐positioned location in close contact with tumor cells and their higher cytotoxic capacities explain why their tumor infiltration is correlated with good clinical outcomes in many cancers. Accordingly, it appears that eliciting Trm represents a key target for the success of cancer vaccines.

The phenomenon of antigen spreading, which corresponds to the secondary CD8^+^ T cell response against antigen epitopes that are not present in the vaccine but likely result from local release after the first wave of tumor‐specific CD8^+^ T cells, provides additional evidence for the important role of these effector cells.^120^ Indeed, in human clinical case studies, it was reported that tumor regression may be dependent on CD8^+^ T cells directed against tumor antigens not present in the vaccine, and thus, the induction of CD8^+^ T cells might be a suitable surrogate biomarker based on immune‐related response criteria to evaluate therapeutic vaccines.^121^

One of the most critical issues for efficient priming of naïve CD8^+^ T cells relates to their contact with mature antigen‐presenting DCs. Some vaccine formulations including DNA‐, RNA‐, or DC‐based vaccines exhibit direct immunostimulatory properties and have been shown to elicit CD8^+^ T cells in mice and in humans.^122^ However, some discrepancies between results obtained in mice and humans, especially for DNA vaccines, have to be addressed.^123^ The plasmid‐based DNA vaccines were thus very efficient in many species including mice and non‐human primates, but early human studies failed due to low efficacy. The plasmid technology has greatly improved over the years, both when it comes to codon optimization increasing expression of the antigens, and plasmid delivery within the body. So, it will be interesting to follow these techniques in the future.

Immunostimulatory adjuvants such as TLR ligands, CD40 agonists, cytokines, or activators of stimulator of IFN genes (STING) are indicated to potentiate vaccine immunogenicity and to break self‐TAA tolerance.^124^ Interestingly, some adjuvants can also preferentially polarize the immune cells to a Tc1‐type CD8^+^ T cell response. For example, vaccination with CpG and Poly I:C increased the ratio between effector CD8^+^ T cells and T_REG_.^125^, ^126^ Activators of STING delivered via a particle platform to reach the cytosol also favor the induction of CD8^+^ T cells.^127^ To date, relatively few immunostimulants have been approved for human use, although several are under evaluation in clinical trials.^128^

One of the most promising strategies for development of therapeutic vaccines against tumor targets uses recombinant, attenuated live vectors derived from viruses or bacteria as a vehicle for the tumor target antigen. Because of their intrinsic capacity to reach the cytosol of the cells, these recombinant pathogen‐derived delivery systems favor MHC class I‐restricted peptide presentation and generally induce a better CD8^+^ T cell response than free proteins or peptide vaccines.^129^ In addition, they are highly immunogenic as they express PAMPs and can be engineered to also express activating molecules like cytokines (IL‐2) or immunomodulatory molecules (B7.1; or a triade of costimulatory molecules called TRICOM: B7.1, ICAM‐1, and LFA‐3) to further amplify the vaccine response.^130^ However, it has to be considered that the efficacy of live vectors can be reduced after repeated vaccinations due to anti‐vector antibody responses. A diversified, prime‐pull strategy using distinct attenuated pathogens is required to overcome the neutralization of the vector bacteria or virus by host immunity. A recently described deep learning approach to neoantigen identification^131^ offers promise for more rapid and targeted tumor antigen discovery, and novel adjuvant and delivery strategies are being evaluated. Several non‐replicating live vectors have been tested in clinical trials with minimal toxicity and the ability to generate antigen‐specific CD8^+^ T cells.^132^ Adjuvant systems such as ISCOMS, saponins, emulsions, liposomes, virosomes, or nanoparticles (Table 3) can also favor cross‐presentation to CD8^+^ T cells in parallel with delivering immune signals. Allostatine is a short peptide derived from Alloferons, a group of naturally occurring peptides isolated from bacteria‐challenged larvae of the blow fly *Calliphora vicina*. These larvae are well known for medical use in wound healing: Napoleon's army was using them on wounded soldiers (a.k.a. “Surgical maggots”). Allostatine is a synthetic linear peptide having amino acid sequence HGVSGWGQHGTHG. It demonstrated strong adjuvant properties in a mouse P388/DBA2 tumor transplantation model when combined with a vaccine consisting of X‐ray inactivated tumor cells.^133^ Although the vaccine alone demonstrated only weak tumoristatic effect in a quarter of the recipients, its combination with allostatine caused a tumoristatic effect in 65% of recipients and prevented tumor appearance in another 30% (the overall positive effect was 95%). The anti‐tumor effect of allostatine "alone" (tumor‐free animals two months after tumor implantation) has been detected in only 9% of the animals. Alloferons are antiviral and anti‐tumor peptides isolated from insects, exhibiting stimulatory effect on the cytotoxic activity of human and mouse NK cells.^134^, ^135^ The Ig site is an evolutionarily conserved pattern in all human and mouse immunoglobulins. Even very low concentration of allostatine in culture medium (at ng/ml scale) causes rearrangement of NK and T cell receptors, stimulation of NK cell cytotoxic activity against cancer cells, and an increased number of IFN‐γ and IL‐2‐producing cells. Allostatine is non‐toxic to immune cells, even at high dose, water soluble, stable in aqueous solutions and can be produced in large quantities at relatively low cost.

**FIGURE 4:**
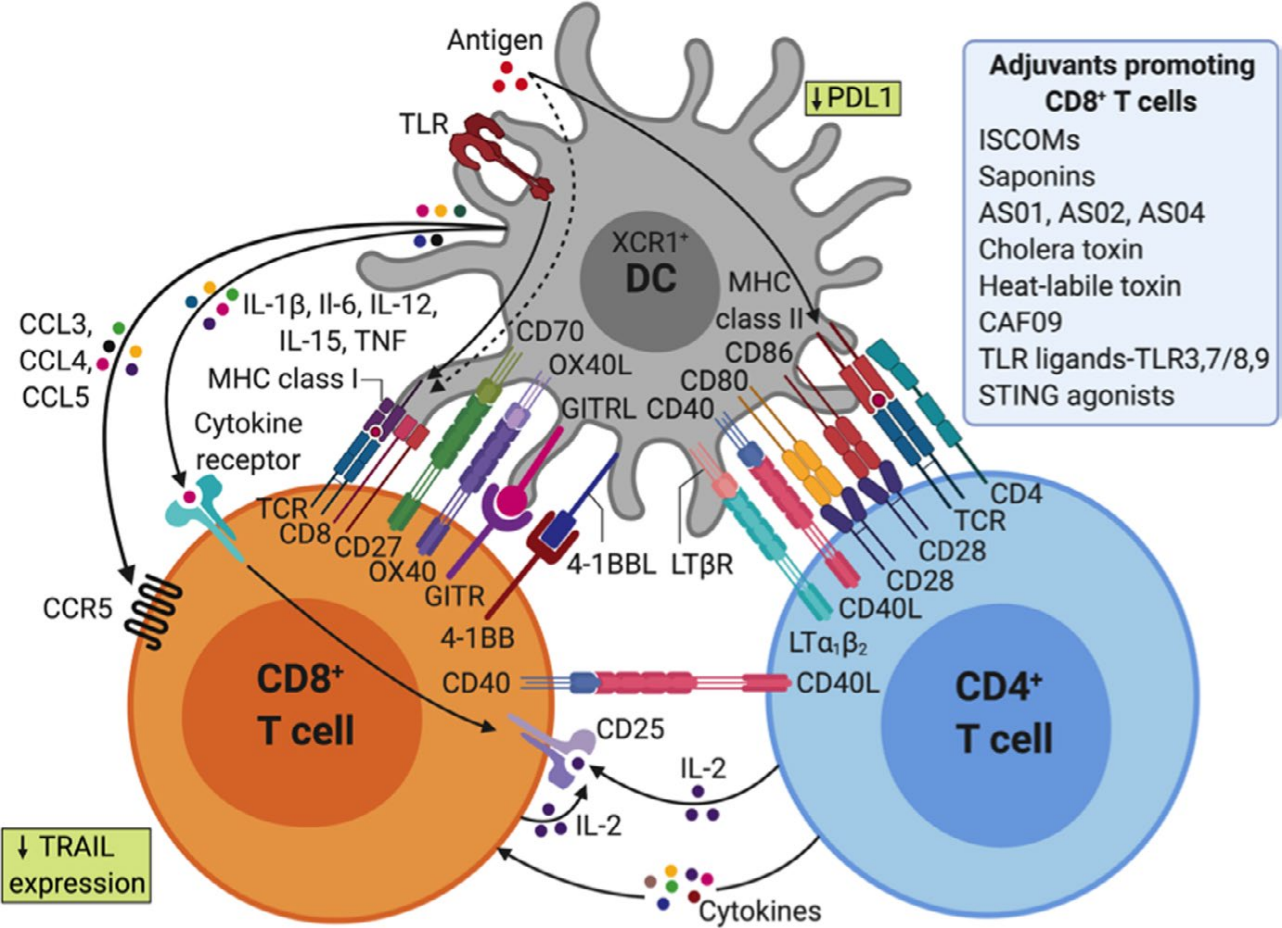
Adjuvants shape CD4^+^ and CD8^+^ T cell immunity through direct and indirect effects on dendritic cells (DCs)

Short CD8^+^ peptides can bypass the need for cross‐presentation by directly binding to MHC‐I molecules on the surface of the APC, but they show low efficiency due to the lack of CD4^+^ T cell help, illustrating the importance of cross‐presentation. It is well‐established that CD4^+^ T cells and especially Th1 cells play a key role in promoting anti‐tumor cellular immunity, mediated via their “helper” function.^136^ Activation of CD4^+^ T cells is essential for optimal CD8^+^ T cell priming, by enhancing antigen presentation and favoring cross‐priming of tumor antigen by DC^137^ (see also Figure 4). CD4^+^ T cells also support recruitment of CD8^+^ T cells to the tumor site, maintenance, and expansion of a CD8^+^ T cell memory response.^138^ Besides their “help” to CD8^+^ T cell responses, CD4^+^ T cells can mediate other anti‐tumor effects such as direct killing of tumor cells, recruitment toward and activation of innate immune cells (eg, NK cells or M1 macrophages) at the tumor site, and modulation of the tumor microenvironment by anti‐angiogenic effects.^139^, ^140^ CD4^+^ Th1 cells also promote vessel normalization leading to better intratumoral CD8^+^ T cell infiltration.^141^

Certain DC subpopulations, especially human CD141^+^ DC designated as cDC1, are thought to be specialized for cross‐presentation.^142^ Recent studies revealed that antibody‐based targeting of antigens to specific DC receptors can enhance antigen uptake and anti‐tumor vaccine efficiency.^143^ Alternative targeting strategies are also possible based on the fusion of tumor antigen(s) to a protein that can engage with receptors on the DC membrane in situ. For example, the group of Eric Tartour demonstrated that targeting DC in vivo with the B subunit of Shiga toxin (STxB), a non‐toxic vector, which binds to Gb3, preferentially expressed by DCs, significantly increased antigen‐specific CD8^+^ T cell responses when coupled to various antigens.^144^ From this pioneering study, a number of cancer vaccines have been developed based on the targeting of surface molecules preferentially expressed on DC (DEC‐205, Clec9a/DNGR, XCR1, CD11c).^145^ Antibodies against human DC receptors such as DEC‐205 and Clec9a coupled with model antigens reported an increase of cross‐presentation, resulting in enhanced induction of antigen‐specific CD8^+^ T cells.^146^ Alternatively, tumor antigen may be formulated and administered ex vivo to DCs in the form of a cell therapy product.^147^ However, the choice of the subpopulation of DC to be selected, the maturation signal to be used and the optimal formulation of antigen remain a matter of debate.^148^ While this type of cancer vaccine has been shown to induce specific CD8^+^ T cells, it has had variable clinical impact,^149^ although the approach remains under evaluation. In this context, specific adjuvants may have potential to optimally activate DC in vitro before adoptive transfer into patients.

The ideal setting for treatment with a therapeutic anti‐cancer vaccine is after surgical resection and/or chemo/radiotherapy, where the induction of anti‐tumor immunity can contribute to eliminate residual circulating cancer cells and micrometastasis. However, despite extensive efforts and promising preclinical studies, therapeutic cancer vaccines have shown limited objective tumor regression and therefore have mostly proven unsuccessful in the clinic. This may be explained by the state of immune suppression after chemotherapy and radiotherapy in the patient and the escape from immune surveillance of newly mutated tumor cells. These anti‐cancer vaccines have been hampered by poor immunogenicity and limited triggering of rare tumor‐specific immune cells, which makes the elicitation of both humoral and cellular immunity challenging and in most cases unsuccessful.^150^

The range of tumor‐associated antigens recognized by T cells provides a variety of potential targets for cancer immunotherapy. In the case of TAAs targeted via cell‐mediated immunity, the activated T cells induced by vaccination recognize the MHC‐peptide complexes of the tumor antigen on the cell surface and not the surface protein itself. Thus, the optimal tumor‐associated antigens for triggering cellular immune responses are not necessarily cell surface proteins and can be classified in several categories^151^:

- Cancer‐testis antigens—normally expressed in germ cells but aberrantly expressed in tumor cells: BAGE, GAGE, MAGE (in melanoma, bladder cancers), and NY‐ESO1 (in melanomas and ovarian cancers; antibody and T‐cell responses have been observed in patients).
- Differentiation antigens—expressed on tumor cells and normal cells from the same tissue: Melan‐A/Mart‐1, gp100, tyrosinase (in melanomas), PSA (in prostate cancer), and CEA (colon).
- Overexpressed antigens—expressed on normal cells at low levels, but at higher levels on tumor cells: HER2 (breast), hTERT, p53, survivin (melanoma), and MUC1 (breast, prostate, colon).

Tumor‐associated peptides antigens, in particular MHC class I‐restricted peptides derived from cancer‐testis and differentiation antigens, have been used in cancer vaccine clinical trials as a means to generate anti‐tumor T‐lymphocyte responses. These vaccines have generated some cytotoxic T‐lymphocyte (CTL) activity; however, the frequency and duration of these responses have been uniformly low and the clinical outcomes have been limited.^150^ Accordingly, the design of therapeutic vaccines against cancer, especially for treating early disease, should not focus only on skewing a response to a single T‐helper cell type (for instance, Th1) or one effector mechanism (for instance, CTL). Rather, the ideal cancer vaccine should seek the simultaneous activation and synergy of both arms of the immune system to generate as many effector cells as possible to eradicate the tumor. Instead of using single peptide, protein, or carbohydrate antigens as TAA for cancer vaccines, cancer antigens prepared from surgically removed autologous or allogeneic tumor tissue may provide a source of TAAs with all, or many, of the required antigens in one preparation.^152^, ^153^

Despite extensive efforts and clinical trials with vaccines targeting TAAs, the clinical success of anti‐cancer vaccine therapies remains limited, with the DC‐based prostate cancer vaccine Sipuleucel‐T being the first and only human therapeutic cancer vaccine approved by the US Food and Drug Administration (FDA).^154^ To advance clinical development of cancer vaccines, further efforts are required, not only to optimize and discover new tumor‐associated antigens, but also to identify and develop improved vaccine adjuvants, delivery systems, and formulations, building upon an increased understanding of the mechanisms underlying the induction of the required immune responses. Moreover, the recent success of checkpoint inhibitors has set the stage for combination strategies and opened the door to revisit cancer vaccine design. Taken together, progress on this front will enable realization of the full potential of a patient's immune response to target and combat cancer.

### 3.3 Atherosclerosis vaccines

Although promising results, as mentioned earlier in this paper, suggest an important role for LDL‐specific antibodies in clearing modified LDL particles and reducing atherosclerosis development; not all ApoB100 peptides elevate LDL‐specific antibody titers after vaccination. In fact, an increasing body of evidence points to a role for T cells in mediating the protective effect of LDL vaccination. Transfer of CD8+ T cells from alum‐ApoB100 peptide vaccinated mice reduced atherosclerosis in the recipient mice, suggesting that CD8+ T cells play a protective role, potentially by cytolysis of antigen‐presenting cells. However, more literature points to a crucial role of regulatory T cells and the induction of immunological tolerance toward LDL. Continuous exposure to ApoB100 peptides with an osmotic pump, mucosal vaccination with ApoB100 peptides covalently linked to cholera toxin B subunit, or oral vaccination with oxidized LDL induced IL‐10 producing regulatory T cells, which have previously been reported to be atheroprotective. These results strongly suggest that vaccine strategies should avoid overt inflammation and should aim at induction of regulatory T cells. Kobiyama and colleagues showed that the adjuvant, AddaVax (a squalene emulsion‐based adjuvant), can replace CFA and reduce atherosclerosis and that production of IL‐10 may be achieved by appropriate adjuvant selection. Specifically aiming at induction of regulatory T cells, Benne and colleagues showed that vaccination with tolerogenic liposomes containing ApoB100 peptides can reduce atherosclerosis and inflammation inside the lesion. Hence, currently it is expected that further development of adjuvants specifically designed for induction of tolerance or regulatory T cells may greatly benefit vaccination against atherosclerosis.

## 4 THERAPEUTIC VACCINES TO MODULATE CELLULAR IMMUNE RESPONSES FOR INFECTIOUS DISEASES

### 4.1 Therapeutic vaccines targeting human immunodeficiency virus

Human immunodeficiency virus (HIV) therapeutic vaccine candidates are designed to induce protective immune responses that control viral replication in the presence or absence of anti‐retroviral therapy (ART). To date, no candidate has proven successful in a controlled randomized trial to achieve long‐term HIV remission.^155^ The low efficacy of many documented strategies appears to result from their inability to induce a broad immune response which suppresses the diverse escape variants that emerge during viral rebound and to purge the HIV reservoir. However, recently published combinatorial approaches demonstrated a high degree of efficacy following anti‐retroviral therapy interruption (ATI) with highly potent antiviral activity demonstrated in non‐human primates (NHP).^156^, ^157^

Most of the unsuccessful candidates were designed to stimulate HIV‐1 gag‐specific CD8^+^ T cell responses as this has been associated with virologic control in preclinical models and in long‐term non‐progressors (LTNP). For example, the use of a recombinant adenovirus serotype 5 (Ad5) expressing the Gag antigen in HIV‐positive patients under ART was unable to induce a strong T cell response and to control viral rebound after ATI (anti‐retroviral therapy interruption).^158^ Also, the use of a MVA poxvirus (MVA.HIVconsv) expressing 14 conserved regions of HIV proved poorly immunogenic and did not impact the viral reservoir.^159^ The use of such therapeutic vaccines may be more efficient when combined with drugs able to reactivate the HIV reservoir. Similarly, candidate vaccines designed to induce neutralizing Ab response have not been successful. In the TUTI‐16 trial, a therapeutic vaccine targeting a conserved Tat B‐cell epitope proved unable to block viral rebound after ATI.^160^

Current therapeutic approaches aim to elicit a broader immune response to target escape variants which emerge during viral rebound. The use of autologous dendritic cells pulsed with heat‐inactivated HIV (DC‐HIV) in viremic untreated HIV^+^ patients induces broad T‐cell responses, resulting in a significant decrease in viral load.^161^ Combination of the poxvirus‐based ALVAC‐HIV (expressing Env, Gag, Pol, and Nef) with Lipo‐6T (Nef, Gag, and Pol peptides combined with a tetanus toxoid peptide and a lipid tail), followed by interleukin‐2, significantly decreased viral rebound after ATI.^162^ Polyfunctional T cell responses were identified as correlates of efficacy. Very recently, it has been confirmed that the addition of IL‐2 with a therapeutic vaccine could reverse T‐cell anergy and increase Ag‐specific T cell responses.^163^ Combination of the DNA‐based vaccine GTUMultiHIV B‐clade with the lipopeptide vaccine Lipo‐5 as well as an MVA‐based vaccine is currently under investigation at the French National Institute for Health and Medical Research‐French National Agency for Research on AIDS and Viral Hepatitis (Inserm‐ANRS).^164^

Hence, from the results obtained in the different randomized clinical trials, a broad immune response seems to be required to have an effect on viral variants appearing during the viral rebound. A simian immunodeficiency (SIV) vaccine, based on a rhesus cytomegalovirus vector (rhCMV) expressing Gag, has been described by the group of Picker.^165^ This vaccine induced an effector memory T cell response that cured 50% of chronically infected monkeys. Follow‐up studies showed that rhCMV elicited an unexpectedly broad T cell response.^166^ Unconventional T cell responses could also contribute to the therapeutic efficacy of rhCMV, with SIV‐specific CD8 T cells recognizing peptides in the context of non‐classical MHC‐E (HLA‐E) molecules. Despite the high efficacy of this strategy, moving this approach to a human trial remains a challenge as human CMV is pathogenic and also because it is unknown whether such non‐classical HIV‐specific CD8 T‐cell responses could be induced in humans.^167^

Recent promising studies, described in non‐human primates on ART, also demonstrated the need for a combination of broad therapeutic vaccines with the activation of innate immunity. A combined approach with an adenovirus serotype 26 (Ad26) and MVA vectors (both expressing Env, Gag, and Pol), with or without a toll‐like receptor 7 (TLR7) agonist, in SIV‐infected monkeys on ART proved to be very efficient.^168^ The Ad26/MVA therapeutic vaccine regimen strongly increased both the magnitude and the breadth of T and B cell response. Interestingly, only the combination of Ad26/MVA with a TLR7 agonist exhibited significant reduction in viral load and a delay of viral rebound. Notably, 33% of the monkeys showed a sustained undetectable viral load following ATI. Ad26/MVA vaccine is currently being tested in HIV^+^ patients who initiated ART during acute HIV infection in Thailand and in the setting of chronic HIV infection at the Beth Israel Deaconnes Medical center (BIDMC). The combined approach with the TLR7 agonist will be also tested in clinical trials. Alternatively, the combination of therapeutic vaccines with existing check point inhibitors, blocking for example PD1 or CTL4, could be very potent approaches. Currently, many promising randomized clinical trials have been designed on these concepts and are under investigation during ATI phases. Thus, therapeutic vaccines able to induce broad immune responses (polyfunctional T cells and broad neutralizing antibodies) carry the potential to strongly impact the viral rebound during ATI.

### 4.2 Therapeutic vaccines against tuberculosis

The only licensed vaccine for tuberculosis (TB) today is a prophylactic vaccine. *Bacille Calmette Guérin* (BCG), an attenuated strain of *Mycobacterium bovis,* is moderately effective in preventing TB in infants/young children, while being unreliable in preventing pulmonary TB in adolescents and adults. As of early 2018, there were 13 candidates in the clinical vaccine development pipeline, which can be classified into the following categories: (a) prophylactic pre‐exposure (priming) vaccines, administered to neonates prior to first exposure to *M tuberculosis* (Mtb); (b) prophylactic postexposure vaccines, given to adolescents/adults with latent TB infection having been previously immunized with BCG, and (c) therapeutic vaccines, intended for administration as an adjunct to conventional TB therapy, to a population at higher risk of developing recurrent disease.^169^ The major goal of therapeutic TB vaccines is to boost the cellular immune response (Th1 type) against Mtb, to shorten the treatment and improve outcomes, such as reducing treatment failure and preventing recurrence, particularly in multi‐drug resistant TB. Several vaccine candidates are currently under development as therapeutic vaccines, including inactivated whole‐cell vaccines, which do not require co‐administration with an adjuvant, while the recently emerging subunit vaccines capable of acting as prophylactic as well as therapeutic vaccines are usually adjuvanted.

A heat‐inactivated whole‐cell vaccine derived from *M vaccae* (named Vaccae), in an injectable form, has already been approved in China for the adjunctive treatment of TB.^170^ It should be noted that a comprehensive review of results obtained in phase II and III trials also supports the use of multiple oral doses of Vaccae (formulated as a tablet) as an adjunct to conventional TB therapy. It not only boosted the Th1 response but also inhibited the Th2 response, thus enhancing host defense against Mtb and demonstrating a marked therapeutic potential.^171^, ^172^, ^173^ The TB vaccine, named RUTI, on the other hand, needs to be administered following previous anti‐TB drug therapy, as it is not able to directly reduce the bacterial load.^174^ Interestingly, RUTI was the first therapeutic vaccine designed to target, and subsequently destroy, the non‐replicating Mtb bacilli via the induction of a Th1 poly‐antigenic cellular response. It is composed of detoxified liposomal cellular fragments of Mtb cultured under stress conditions mimicking those that exist inside granulomas. Under these conditions, latency antigens are induced, which are normally concealed from the immune system, thus facilitating the generation of immune responses against both non‐replicating and replicating Mtb. The results obtained in phase I and II clinical studies in HIV‐positive volunteers with latent infection demonstrated safety and immunogenicity, respectively.^175^ Lastly, another inactivated non‐tuberculous mycobacterial vaccine based on *M indicus pranii* has also been studied in a phase II trial as an adjunct to TB therapy capable of inducing a Th1 response.^176^

Subunit prophylactic/therapeutic vaccine candidates either contain plasmid DNA or fusion proteins presenting a combination of antigens covering both active and latent TB stages or usually require formulation with an adjuvant.^169^ ID93/GLA‐SE is a vaccine candidate containing a fusion protein, specifically a recombinant antigen called ID93, integrating virulence‐associated proteins Rv2608, Rv3619, and Rv360 or latency‐associated protein Rv1813. This recombinant antigen is formulated with the synthetic Toll‐like receptor 4 (TLR‐4) agonist GLA, a glucopyranosyl lipid A in a stable nanoemulsion (GLA‐SE).^177^, ^178^ The latter allows for the induction of a robust Th1 response and has shown promising preclinical results, since it significantly enhanced the efficacy of anti‐tuberculous drugs in a mouse model.^179^, ^180^

A plasmid DNA vaccine encoding mycobacterial heat shock protein 65 (Hsp65) and interleukin‐12 (IL‐12), delivered by the hemagglutinating virus of Japan (HVJ) envelope (E) in liposomes,^181^ induced protective as well as therapeutic efficacy against drug resistant TB in murine models in combination with anti‐tuberculous drugs.^182^, ^183^ The efficacy of this Hsp65 vaccine was further enhanced with other plasmid DNA‐based vaccines encoding the granulysin or Ksp37.^182^ Ag85 complex proteins possessing mycolyl transferase activity are highly immunogenic and are secreted by all mycobacterium species. A vaccine candidate consisting of Ag85AB protein complex emulsified and adjuvanted with a TLR9 agonist (CpG‐ODN) and dimethyldioctadecylammonium bromide elicited a strong Th1 response when co‐administered with anti‐tuberculous drugs.^184^ A similar therapeutic efficacy has been achieved by DNA vaccine candidates consisting of plasmid DNA encoding either Ag85A or Ag85B which enhanced the efficacy of concomitantly administered anti‐tuberculous drugs via the augmented secretion of Th1‐type cytokines and a suppressed release of Th2‐type cytokines in preclinical trials.^185^, ^186^, ^187^ Recent data demonstrate that the adjuvanted subunit tuberculosis vaccine M72/AS01E is immunogenic in TB‐infected patients^188^ offering promise for the development of a more effective therapeutic TB vaccine.

## 5 THERAPEUTIC VETERINARY VACCINES

While veterinary therapeutic vaccines have a rather long history, relatively few approaches have resulted in commercial products, and most of them originated from human medical platforms. Initial attempts focused on oncological diseases in production animals. In 1958, an autologous vaccine was developed for bovine papillomatosis, a viral disease of cattle characterized by the presence of mucosal and cutaneous neoplastic lesions. It was synthesized from formalin‐inactivated supernatants of affected tissue.^196^ The autologous vaccination procedure reportedly induced remission of the skin lesions in diseased cattle.^196^, ^197^ However, the results were based on clinical observations, and experimental data are lacking from the literature. Recent publications showed high recovery rates, using therapeutic schemes related to human papilloma virus vaccines, but none of them reached the market (reviewed by 198).

The causative agent of bovine papillomatosis, the bovine papilloma virus, is also involved in the pathogenesis of equine sarcoid, the most frequent skin tumor in horses.^199^ Several research groups investigated the efficacy of autologous vaccines against equine sarcoids.^200^, ^201^, ^202^ According to these publications, complete tumor regression was noted in a high percentage of affected horses. However, the number of studied cases was limited, while other strategies, such as chemotherapy or surgical removal of the lesions, have been suggested as alternative treatments.^203^ The current absence of commercially registered therapeutic tools favors the development of small private laboratories, which are specialized in the production of autologous vaccines, not only for equine sarcoids but also for other malignancies of companion animals.

In contrast to the human medical sector, the pharmaceutical industry seems hesitant to heavily invest in veterinary anti‐cancer immunotherapeutics, with the exception of the canine oral melanoma vaccine, Oncept (Merial, Duluth, GA, USA). Oncept, primarily based on studies for human vaccines, activates an immune response to tyrosinase, an enzyme overexpressed in melanoma tumor cells, which is usually ignored by the dog's immune system. Vaccination with Oncept triggers the immune responses toward a protein expressed by a xenogenic human tyrosinase gene, incorporated into a plasmid vector.^204^ Clinical trials in dogs with surgically resected tumors revealed significantly prolonged survival times,^204^, ^205^ although in later studies a clear correlation between immunological parameters and loco‐regional cancer control could not be established.^206^

The recently licensed Cytopoint (Cytopoint, Zoetis, USA), a monoclonal antibody‐based immunotherapeutic against atopic dermatitis in dogs, illustrates the growing demand for novel products in the field of companion animal welfare. Apart from animal health‐specific issues, effective therapeutic veterinary vaccines will have a strong impact on public health through reductions in the transmission rates of potential zoonoses. For example, a vaccine against dermatophytosis (Insol Dermatophyton, Boehringer Ingelheim, Germany) also known as ringworm, a disease that can be transmitted between animals and humans, has been approved in many European countries for therapeutic or prophylactic use in dogs, cats, and horses. Insol dermatophyton is reported to accelerate the healing of skin damage caused by the fungus. A similar therapeutic effect was demonstrated particularly for cats, after administration of an inactivated vaccine containing strains of *Microsporum canis*, *Microsporum gypseum,* and *Trichophyton mentagrophytes*.^207^

Since the field of animal vaccinology has more flexible regulatory and preclinical trial requirements, significant knowledge regarding the safety and the effectiveness of the vaccine in the target animal can be achieved relatively quickly. In addition, insights into adjuvant mechanism of action in this context could then inform studies on other vaccines with benefits for both animal and human indications. However currently, details regarding the immunomodulatory adjuvant components in these vaccines are often not publicly available, being protected as proprietary information by the vaccine manufacturer.

## 6 FUTURE PERSPECTIVES

Since a majority of chronic diseases affecting humans and animals have an underlying immunological basis, therapeutic vaccine approaches can be envisaged to intervene, to eliminate or reduce disease symptoms. As a result, therapeutic vaccination has great potential to address a range of infectious and non‐infectious conditions. However, there are many challenges in comparison with prophylactic vaccination including disease heterogeneity, a requirement to block or divert ongoing immune responses, and a lack of clarity on the optimal antigens.

Progress in this field will depend on developing novel adjuvant approaches and appropriate tailoring of adjuvants to achieve a specific goal. Given the enormous diversity in requirements from driving potent tumor‐specific T cells in the case of cancer to a dominant Treg response for autoimmune diseases, the types of adjuvant required will be diverse and it would be a major step forward if a panel of clinically acceptable adjuvants that can achieve these diverse outcomes were made available. While there are very promising advances in antigen discovery, exciting recent developments, particularly in situ vaccination for cancer,^208^ offer the potential for adjuvant strategies to act as therapeutics without the challenge of identifying and incorporating specific antigens. Related to this, innate immune training presents strategies to modulate innate immunity to drive effector responses or reduce inflammation in an antigen‐independent manner and strategies toward identifying the optimal adjuvants to achieve this are being evaluated.^209^

In the case of cancer (and autoimmune diseases), it is likely that vaccination approaches will be used in parallel with other therapeutic strategies. In particular, for cancer vaccines, optimal combinations of therapeutic vaccines and checkpoint inhibitors are likely to be more effective than the vaccines alone. In the case of autoimmune diseases, therapeutic vaccines that reduce inflammatory T cell responses could be combined with anti‐cytokine therapies to bolster anti‐inflammatory responses and reduce pathology. Overall, although this is a challenging field, the future appears bright and there will be a major role for adjuvant researchers to facilitate the generation of innovative new therapeutic vaccine strategies.

## CONFLICT OF INTEREST

Jens Brimnes is an employee at ALK‐Abello A/S, a company that manufactures and sells allergy immunotherapy (AIT) products. Sergey Chernish is the owner of Alopharm, a company producing Allostatines. Virgil Schijns is shareholder and Chief Scientific Officer at Epitopoietic Research Corporation (ERC), a company producing an immunotherapy against glioma brain cancer. All other authors declare no competing interest.
